# Current understanding of mdig/MINA in human cancers

**DOI:** 10.18632/genesandcancer.73

**Published:** 2015-07

**Authors:** Chitra Thakur, Fei Chen

**Affiliations:** ^1^ Department of Pharmaceutical Sciences, Eugene Applebaum College of Pharmacy and Health Sciences, Wayne State University, Detroit, MI, USA

**Keywords:** mdig/MINA, human cancers, immune regulation, disease prognosis, biomarker

## Abstract

Mineral dust-induced gene, mdig has recently been identified and is known to be overexpressed in a majority of human cancers and holds predictive power in the poor prognosis of the disease. Mdig is an environmentally expressed gene that is involved in cell proliferation, neoplastic transformation and immune regulation. With the advancement in deciphering the prognostic role of mdig in human cancers, our understanding on how mdig renders a normal cell to undergo malignant transformation is still very limited. This article reviews the current knowledge of the mdig gene in context to human neoplasias and its relation to the clinico-pathologic factors predicting the outcome of the disease in patients. It also emphasizes on the promising role of mdig that can serve as a potential candidate for biomarker discovery and as a therapeutic target in inflammation and cancers. Considering the recent advances in understanding the underlying mechanisms of tumor formation, more preclinical and clinical research is required to validate the potential of using mdig as a novel biological target of therapeutic and diagnostic value.

**Summary:**

Expression level of mdig influences the prognosis of several human cancers especially cancers of the breast and lung. Evaluation of mdig in cancers can offer novel biomarker with potential therapeutic interventions for the early assessment of cancer development in patients.

## INTRODUCTION

Human beings are frequently exposed to chemical and physical hazards like automobile exhausts, smoke and industrial emissions in environmental and occupational settings. Transportation related air pollution, waste incineration, burning of wood, lifestyle and dietary habits like smoking, intake of grilled and charred food are the common sources of toxic agents such as polycyclic aromatic hydrocarbons and its derivatives. These toxic chemicals are the established risk factors for several human cancers [1]. Gene-environment interaction is an important aspect contributing to carcinogenicity and studies pertaining to genes that are responsible for neoplasias upon exposure to environmental insults, is extremely critical.

Mineral dust-induced gene (*mdig*) is one such gene that is environmentally induced and is overexpressed in human cancers. The necessity in exploring more about the in-depth mechanisms of cancer development due to environmentally induced *mdig* gene has become imperative. This review provides the detailed insight about the implication of mdig in cancers, thereby allowing researchers and health care professionals to assess the potential risk factors for human cancers in context to occupational and residential environments.

### Mdig gene: discovery, structure and distribution

In order to understand the molecular basis of the occurrence of pulmonary diseases like lung inflammation, fibrosis, Chronic Obstructive Pulmonary Disease (COPD), or cancer associated with environmental or occupational exposure to mineral dusts, investigators identified a novel gene called as mineral dust-induced gene *mdig* (GenBank BE441202, 2000; AY302110, 2006) as this gene was differentially expressed in alveolar macrophages of coal miners exposed to mineral dust. In-depth analysis of mdig in cell culture system revealed that when human lung cancer cells A549 were exposed to silica particles, it resulted in the induction of mdig mRNA in a time and dose dependent fashion. Interestingly, human lung cancer tissues showed the expression of full-length mdig mRNA but not in the adjacent normal tissue. This was the first study indicating the relation of mdig with respect to mineral dust exposure, which shows the possible implication of this novel gene in cell growth regulation and in human lung cancer [2]. Subsequently, mdig gene was also discovered independently in human promyelocytic leukemia HL60 cells or brain tumor T98G cells with c-myc overexpression and hence named myc-induced nuclear antigen with a molecular weight of 53 kDa (MINA53) [3]. An alternative name, nuclear protein 52 (NO 52) for mdig was given in the literature owing to the fact that mdig protein was found to be localized predominantly in the nucleoli of cells [4]. Hereafter we would denote mdig as mdig/MINA throughout this paper.

The human mdig/MINA gene is located on chromosome 3 (3q12.1) that encodes a protein with molecular weight of 53 kDa, and localizes in the nucleus whereas part of the protein is concentrated in the nucleolus. It consists of 12 exons spanning a 30 Kb region with the coding sequence consisting of 1398 bp encoding 465 amino acids.

### Mdig/MINA: protein structure and characteristics

**Figure 1:**
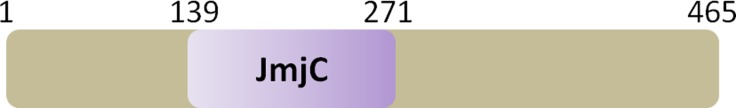
Mdig/MINA protein with the position of JmjC domain shown in purple

The mdig/MINA protein consists of 465 amino acids with an estimated molecular weight of 53 kDa. There are four isoforms of mdig/MINA protein produced due to alternative splicing. Isoform 1 has been chosen as the ‘canonical’ sequence [2, 3, 5-7]. Isoform 2 with a mass of 31.8 kDa [3], isoform 3 with a mass of 23.9 kDa [2] and isoform 4 with a mass of 52.6 kDa has been reported in the literature. Mdig/MINA belongs to the JmjC family of proteins containing one JmjC domain (Figure 1). JmjC has been shown to function in a histone demethylation mechanism and is conserved from yeasts to humans. Proteins containing JmjC domain are implicated in the regulation of chromatin remodeling and are predicted to be metallo-enzymes adopting the cupin fold. Using electro-spray ionization-mass spectrometry guided disulphide cross-linking technology, substrate complexes for mdig/MINA were obtained. MINA (Tyrosine 209C) residue readily cross-links and crystallizes in complex with RPL27A (G37C) [8].

### Mdig/MINA: expression, catalytic activity and induction

**Figure 2:**
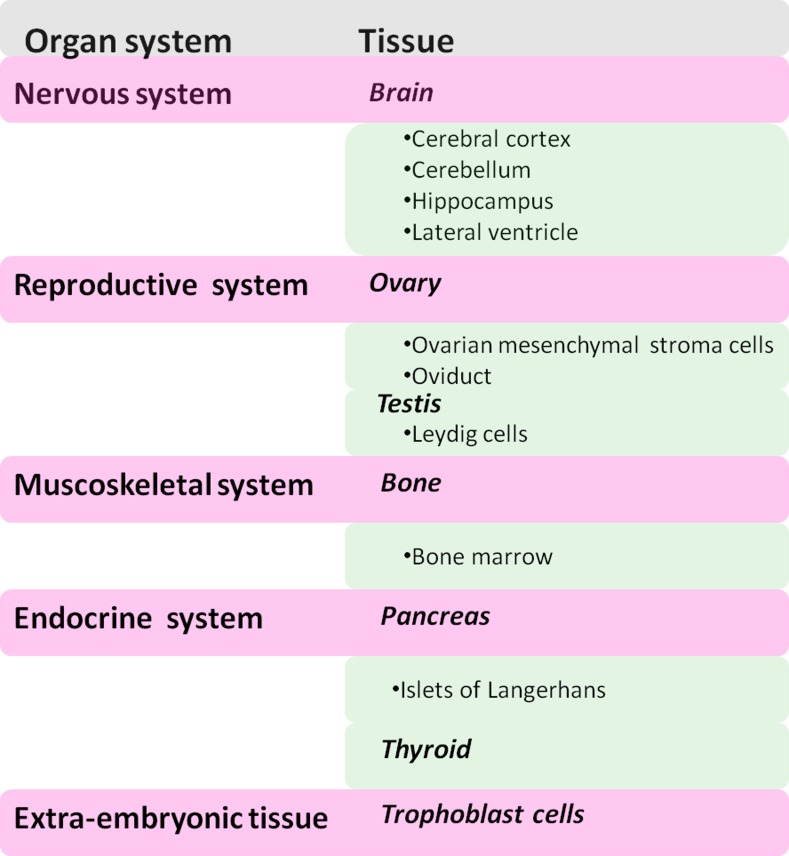
Distribution of mdig/MINA in human tissue

**Figure 3:**
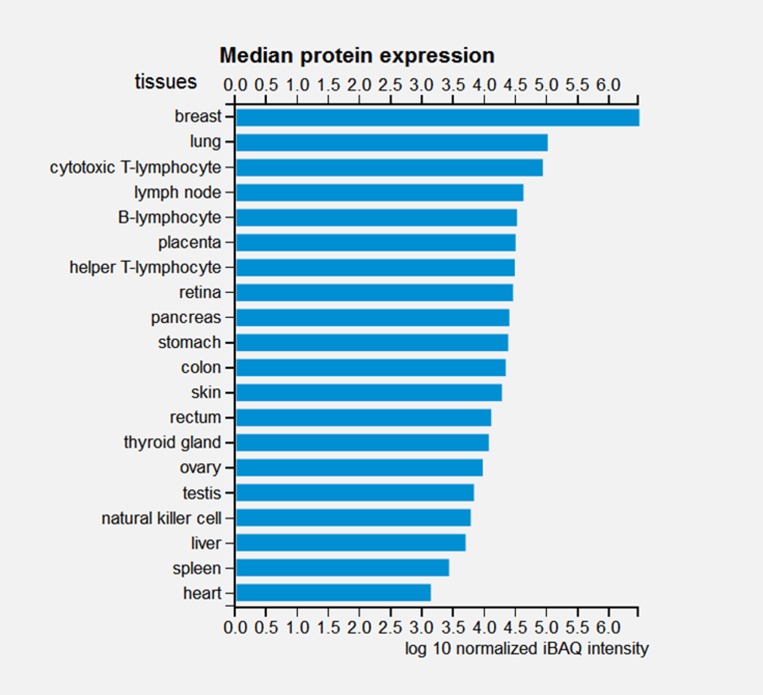
Mdig/MINA expression in human tissues revealed by mass spectroscopy-based analysis of the human proteome Source: ProteomicsDB [9].

Mdig/MINA is ubiquitously expressed and its expression is seen especially in the tissues of endocrine function and immune response. In mice, tissues like spleen, thymus, colon, pancreas and testis exhibit high mdig/MINA expression whereas skeletal muscle, cerebellum and seminal vesicles show low mdig/MINA expression while lung, heart and kidneys have no mdig/MINA expression. In humans, mdig/MINA is frequently elevated in various types of cancers. It is also expressed in liver, heart, pancreas, skeletal muscle and placenta whereas absent in lung, brain and kidney. Reports from the Embryonic Development and Stem Cells Database, Lifemap^TM^ unravels the expression of mdig/MINA in several embryonic tissues and stem cell suggesting that mdig/MINA is critical for embryonic development. These include the major organ system as depicted in Figure 2. Mass spectroscopy-based analysis of the human proteome revealed the relative abundance of mdig/MINA in different cells and tissues (Figure 3) [9].

Proteome suggested the presence of mdig/MINA protein in the lung. However reports also indicate the presence of an alternative mRNA splicing of the mdig/MINA gene. Tissue distribution of mdig/MINA mRNA in normal human tissues as revealed by northern blot analysis showed the presence of mdig/MINA in liver, skeletal muscle, heart, pancreas and placenta whereas in brain, lung and kidney it was undetectable. The length of the detected mRNA corresponded to 1.5kb, that represented the fully spliced mdig/MINA mRNA. Additionally, a 0.4-0.5 kb fragment was detected along with it indicating an alternate spliced one. Interestingly, human lung tumor cell lines also showed the presence of an alternative mRNA splicing of the mdig/MINA gene. Mdig/MINA mRNA was detected in fifteen different human lung cancer cell lines, under basal conditions. However one cell line named H441, suggested the presence of an alternative spliced form of mdig/MINA mRNA called as mdig 2 gene (Gen Bank Access no. A4456380) [2].

The orthologs of mdig/MINA gene suggested that it was present in the common ancestors of animals that ranged from the common worm (Caenorhabditis elegans) with the gene named as jmjc-1 and the isoform a of protein JMJC-1 [6], to the chimpanzees in mammals [3]. Mdig/MINA is a ribosomal oxygenase, which has been structurally conserved from prokaryotes to humans [8]. It belongs to a class of enzyme designated as oxidoreductases. It possesses dioxygenases activity and catalyzes the hydroxylation of 60S ribosomal protein L27a on Histidine 39 with the following biochemical equation with Fe^+2^ as a cofactor which binds 1 Fe^+2^ ion per sub unit.

L-histidine-[60S ribosomal protein L27a] + 2-oxoglutarate + O(2) = (3S)-3-hydroxy-L-histidine-[60S ribosomal protein L27a] + succinate + CO(2)

With the findings that ribosomal oxygenases occur in organisms that ranges from prokaryotes to humans, compelled the researchers to think regarding their structural and evolutionary relationships. Using structure-informed bioinformatic analysis, co-immunoprecipitations and NMR studies, investigators identified a new set of 2 OG-oxygenases (2-oxo glutarate dependent oxygenases) mdig/MINA and NO 66 (Nucleolar protein 66). In E.Coli, YcfD is an enzyme that catalyses the arginine hydroxylation in the ribosomal protein L16. It is a growth regulating 2-oxo glutarate oxygenase. Interstingly YcfD is related to human oxygenases. Human YcfD homologs, mdig/MINA and NO66 are also linked to growth and catalyze histidine hydroxylation in the ribosomal proteins Rpl27a and Rpl8, respectively. An Rpl27a fragment was revealed via peptide screening of mdig/MINA interactors. Mdig/MINA catalyzed (2S, 3S)-3-hydroxylhistidine modification of Rpl27a at residue 39, that was identical to the one catalyzed at Rpl8 H2916 by NO 66. ShRNA-mediated knockdown suggested that Rpl27a hydroxylation is mdig/MINA dependent. Whole-protein MS quantification analysis revealed that Rpl27a is more than 90 percent hydroxylated in A549 cells, HEK 293T cells, several murine tissues, normal human placenta and tumor samples from human Hodgkin lymphomas [10].

Mdig/MINA is induced in the alveolar macrophages of individuals working in coal mines. In A549 lung adenocarcinoma cell line, mdig/MINA is induced upon silica treatment [2]. It is also induced in normal bronchial epithelial cells BEAS-2B and A549 cells upon arsenic treatment in a dose and time dependent manner [11]. When serum starved T98G glioblastoma cells were stimulated with serum, there is a five-fold increase in mdig/MINA expression. Induction of mdig/MINA is also detected in differentiating promyelolytic leukemia HL60 cells and the mRNA levels of mdig/MINA correlated with that of c-myc expression [12].

### Role of mdig/MINA gene: in vitro and in vivo functional studies

#### Cell proliferation

The biochemical features of mdig/MINA gene have been elucidated by a variety of cell culture experiments and in vitro analysis. Mdig/MINA is induced in human lung cancer A549 cells when treated with silica in a dose and time-dependent manner with a 2.5 fold induction of mdig/MINA mRNA for 18 hrs silica treatment. SiRNA-mediated RNA interference technique suggested the involvement of mdig/MINA gene in cell growth and silica induced cytotoxicity. Silencing of mdig/MINA mRNA delays cell cycle transition from G1 to S phase resulting in the inhibition of cell proliferation [2]. Oncoprotein c-myc directly induces the expression of mdig/MINA [3]. The growth promoting characteristic of mdig/MINA was additionally confirmed by over expression of mdig/MINA in lung cancer cell line. All mdig/MINA stably expressing clones displayed increased proliferation whereas proliferation was reduced in the cells expressing mdig/MINA shRNA. Interestingly, an enhanced ability of migration and invasion was observed in the cells where mdig/MINA expression was silenced by shRNA indicating an inhibitory effect of mdig/MINA on cell migration and invasion. These data clearly indicated the paradoxical roles of mdig/MINA on cell proliferation, invasion and migration [13].

#### Ribosome biogenesis

Nucleolus is also called as the ribosome factory and one of the essential components of cell proliferation is ribosome biogenesis. Mdig/MINA gene is accumulated in the nucleolus [3] where it is highly concentrated in the granular component of nucleoli. While free peri-ribosomal particles exhibit mdig/MINA, cytoplasmic ribosomes show no mdig/MINA in them. Mdig/MINA interacts with various ribosomal proteins and is involved in the ribosome's biogenesis, prominently during the assembly process of peri-ribosomal particles [4]. It is also involved in the ribosomal RNA transcription [14].

#### Epigenetics

It is well-known that genetic aberrations contribute to tumorigenesis. Emerging evidence in the past decade also suggested important contributions of epigenetic alterations on histone proteins and DNA to carcinogenesis. One of the features of histone demethylases is that most of these enzymes are JmjC domain containing proteins. Interestingly, human mdig/MINA gene encodes a protein containing a conserved JmjC domain which is likely to affect the methylation status of histone proteins. By establishing stably transfected cell lines through overexpressing mdig-GFP protein in A549 cells, we have demonstrated the mdig/MINA's ability in decreasing the heterochromatin conformation of A549 cells and derepressing the transcription of genes present in the tandemly repeated DNA regions [15]. H3K9me3 is an important regulator and epigenetic marker of heterochromatin and exerts a major effect in maintaining the conformation of heterochromatin. We have reported the histone demethylase activity of mdig/MINA. Our biochemical assays shows that immunoprecipitated mdig/MINA from the cells can demethylate H3K9me3 on histone H3 peptide. Mdig/MINA overexpression leads to a 2.8 fold and 3.4 fold increase in c-myc and Jhdm3a expression levels, respectively. Jhdm3a is an H3K9me3 demethylase that might contribute to the demethylation of H3K9me3. Over expression of mdig/MINA is linked to the derepresssion of the paternally imprinted gene H19. Vice versa, silencing mdig/MINA resulted in a 6.2-fold increase of H19 transcript. Moreover the enrichment of H3K9me3 in the H19 gene promoter region was diminished by the overexpression of mdig/MINA. These studies clearly indicate the role of mdig/MINA in mediating the demethylation on histone and the heterochromatin conformation, which might be one of the important factors rendering mdig/MINA gene in tumorigenesis and genomic instability. This has been further strengthened by the fact that increased expression of mdig/MINA as well as H19 has been associated with the poorer survival of patients suffering from lung cancer [15].

#### Oncogenic potential

Mdig/MINA is a c-myc target gene that plays a role in cell proliferation or regulation of cell growth [2, 3]. One of the hallmarks of cancer is abnormal cell proliferation. siRNA knockdown of mdig/MINA expression caused a significant decrease in HeLa cell proliferation [3]. Similar results were obtained in A549 cells that showed knocking down mdig/MINA delays cell cycle transition from G1 phase to S phase. Additionally, 15 out of 19 human lung cancer cell lines showed mdig/MINA expression. These studies clearly indicate the involvement of mdig/MINA in mammalian cell proliferation [2, 3]. Also, studies show mdig/MINA's frequent expression in human lung cancers and its transformation capacity in NIH 3T3 cells [12]. In human cancers, elevated expression of mdig/MINA has been found to be a characteristic feature of colon cancer [16] and esophageal squamous cell carcinoma [17]. Mdig/MINA has also been linked to lymphoma, especially the tumor progression of B cell lymphoma [18]. It is overexpressed in gastric carcinoma and has been associated with tumor proliferation and anti-oncogene inactivation [19]. Mdig/MINA overexpression in renal cell carcinoma suggested it as one of the factors for poor prognosis in this disease [20]. Increased levels of mdig/MINA has been associated with cholangiocarcinoma with clinical significance [21] and aggressive hepatocellular carcinoma showed high mdig/MINA expression [22]. Mdig/MINA also contributes to the initiation or development of human lung cancer and this is achieved by the altering of histone H3 methylation by mdig/MINA protein [14]. Interestingly, it acts as a favorable prognostic marker in early stage lung cancer especially of stage I or squamous cell carcinoma [23]. Higher levels of mdig/MINA correlate with poorer survival of lung cancer patients. It is involved in the regulation of H3K9me3 to influence the heterochromatin structure of the genome thereby affecting the expression of genes that are critical for cell growth and transformation [15]. This suggests that mdig/MINA contributes to genomic instability, thus facilitating the development of cancers. In NSCLC patients, the levels of mdig/MINA are correlated with the time delay to disease progression in clinical settings [11]. The paradoxical effect of mdig/MINA on cell growth and motility has been suggested at the different stages of neoplastic transformation and increased expression of mdig/MINA correlated with poorer overall survival of lung cancer patients and especially for those patients who were found negative for lymph node metastasis [13]. Recently we reported that increased expression of mdig/MINA serves as an important prognostic factor for poorer survival time of breast cancer patients [24]. All these reports undoubtedly indicate that mdig/MINA has an important role in carcinogenesis and it may serve as suitable target for cancer prevention.

#### Signaling pathway

Since mdig/MINA is linked to occupational lung disease and a newly discovered oncogene, the immediate curiosity was to investigate the role of mdig/MINA in mediating the transformation of cells in response to environmental insults. Arsenic is one among such irritant that is widely distributed in drinking water or underground water in some areas of the world. Our in vitro studies revealed that arsenic induced the expression of mdig/MINA and this was partially dependent on JNK and STAT3 signaling pathway. Using chemical inhibitors and siRNA to disrupt the JNK or STAT3 diminished the accumulation of mdig/MINA mRNA and protein in bronchial epithelial cells administered with arsenic. Moreover microRNA-21 (miR-21) and AKT were the downstream effectors of JNK and STAT3 signaling pathways in arsenic induced mdig/MINA expression [11]. miRNA-21 is the first identified oncogenic miRNA (oncomir) targeting many essential tumor suppressors resulting in the aberrant activation of oncogenic protein kinases like AKT via down regulating PTEN, PDCD4 and Spry 2, the known negative regulator of AKT [25].

#### Immune regulation

The role of mdig/MINA in immune function has been recently acknowledged. The differentiation of naïve CD4^+^ T cells into distinct effector cell lineages has been an important component of the adaptive immune system. This lineage comprises of two different effector cell types designated as Th1 and Th2. The Th1 response leads to cell-mediated immunity whereas Th2 response is characterized by the production of IL-4, resulting in the activation of B-cells linked to humoral immunity. Through the transcriptional profiling analysis using QTL approach, researchers identified mdig/MINA as an important genetic determinant and a negative regulator of IL-4 expression in Th2 bias. It was noted that an inverse correlation exist between the naïve helper T cell's capacity to express IL-4 and mdig/MINA expression, with mdig/MINA acting upstream of IL-4 and IL-12. Mdig/MINA represses the transcription from IL-4 promoter and the target site for mdig/MINA's repressive activity lies in the first 140 bp of IL-4 promoter. Further studies suggested that mdig/MINA could be recruited to IL-4 promoter through NFAT (Nuclear factor of Activated T cells) transcription factor [26]. These findings clearly indicate that mdig/MINA is critical for naïve helper T cells for their capacity to produce IL-4, which influences or control the Th2 differentiation and Th2 bias in immune function. Most recently, studies using silicon nanowires technology to deliver siRNA screening library to T cells and subsequent transcriptional profiling coupled with novel computational algorithms indicated that mdig/MINA is an essential regulator for Th-17 cells. Knockdown of mdig/MINA repressed the expression of Th-17 cytokines and transcription factors (RORc, Batf, Irfu) whereas increased the expression of Foxp3, which is a master transcription factor of T regulatory (Treg) cells [27]. Additionally, gene knockout study of mdig/MINA showed decreased IL-17 and increased Foxp3 positive T cells in the naïve T cells after differentiation from the mdig/MINA−/− mice. Also Rorc knockout mice showed a reduced expression of mdig/MINA in their Th-17 cells. These studies clearly indicate the mdig/MINA's role in promoting Th-17 differentiation and inhibiting Foxp3 programs. Another study also indicated that mdig/MINA affects the reciprocal Treg/Th17 balance by favoring the differentiation of Th17 cells. Mdig/MINA facilitates the transcription of Rorc and suppressed Foxp3 when induced by ROR gammat and Batf factors [28]. Hence mdig/MINA is one of the novel targets that regulate Th17/Treg balance and affecting the Th2 bias thereby indicating its potential role in modulating immune-regulatory function that might predispose an individual to immune-pathogenicity prior to tumorigenesis in the context of mdig/MINA-mediated disease etiology.

#### Animal studies

Studies involving mouse model were attempted to elucidate the role of mdig/MINA in non-neoplastic tissues. A study conducted by Mori et al. showed that mdig/MINA plays an important role in allergic asthmatic response by positively regulating the allergic response via IL-4 production. This study also deciphered the expression of mdig/MINA in monocyte/macrophage lineage. Mdig/MINA deficient mice exhibited reduced migration of immune cells, lowered levels of Th2 cytokines IL-4 and IL-5 in response to house dust in an allergic asthma model [29]. Similarly, our mdig/MINA gene knockout studies in a model of experimental silicosis revealed that the deficiency of mdig/MINA ameliorated silica-induced lung fibrosis and the infiltration of macrophages and Th17 cells by altering the balance between Th17 and Treg cells in the lung [30]. Our studies yielded heterozygotic mdig/MINA knockout mice (mdig+/−) only but not the homozygotic knockout mice (mdig−/−) which was otherwise reported in Mori's study that mdig/MINA−/− mice were fertile and reached adulthood. Our reports suggest that the homozygotic deletion of mdig/MINA gene is lethal for embryogenesis. However the discrepancy in mdig/MINA gene knockout mice, fertility & viability mdig+/− vs mdig−/− in Mori's report is very likely due to the use of different recombinant strategies in disrupting the mdig/MINA gene. We replaced the entire region from exon 2 to exon 8 of the mdig/MINA gene with the neocassette to ensure a complete deletion of the mdig/MINA gene as well as the major alternatively spliced isoforms. In Mori et al. report, only exon 2 was replaced. Perhaps some alternatively spliced mdig/MINA mRNA may exist to support embryogenesis. Our studies revealed that the deficiency of mdig/MINA gene attenuated silica-induced lung fibrosis and infiltration of macrophages and Th17 cells. In other words the presence of mdig/MINA gene favors the formation of lung fibrosis induced by silica through promoting Th17 cells. Nevertheless both the in vivo studies pertaining to mdig/MINA deficiency revealed an attenuated immune response in murine airways, with our studies indicating this observation might result from an impaired function of Th17 cells.

### Mdig/MINA and human cancers: evidence from experiments and clinical studies

#### Breast cancer

We have shown that the survival time of breast cancer patients is affected by the expression level of mdig/MINA. Immuno-histochemical detection analysis revealed that 30% of invasive ductal carcinoma (IDC) and 33% of invasive lobular carcinoma (ILC) showed higher levels of mdig/MINA expression. Meanwhile, an inverse correlation between mdig/MINA expression and patient survival, including poorer overall survival (OS), distant metastasis free survival (DMFS), relapse free survival (RFS), and post progression survival (PPS), was observed. In addition, increased expression of mdig/MINA predicted poorer survival of patients with luminal A subtype of breast cancer. Surprisingly, high mdig/MINA expression appears to be a favorable factor for better overall survival of patients who were lymph node positive [24]. In our previous studies using lung cancer cell lines and lung epithelial cell line, we found that mdig/MINA enhanced cell proliferation but repressed migration and invasion in in-vitro assays [13]. This might support the notion that mdig/MINA predicts better survival of patients with lymph node positive, an indication of cancer cell metastasis.

#### Lung cancer

Overexpression of mdig/MINA appears to be a common feature of non-small cell lung cancer (NSCLC). The significant expression of mdig/MINA in lung cancer tissues but not from normal tissues suggests that mdig/MINA possess oncogenic potential and this may be achieved via affecting epigenetic landscape of the genome, such as tri-methly lysine 9 on histone H3, thereby promoting ribosomal RNA synthesis, expression of H19, c-myc, etc. [14]. Other additional studies also indicated that about 62 percent of the patients with early clinical stages of lung cancer showed mdig/MINA overexpression [12]. Emerging evidence suggested an association of mdig/MINA expression and the lung cancer patient survival. Using an online database containing gene profiling information from 2,437 cases of lung cancer, we found that high level of mdig/MINA predicts poorer overall survival (OS) of the lung cancer patients who had no lymph node metastasis or had only possible proximal lymph node metastasis. In contrast, high level of mdig/MINA had no predictive power on the OS of patients with distal lymph node metastasis [13]. This observation is in agreement with our studies regarding the prognostic value of mdig/MINA for breast cancer patients, in which an increased expression of mdig/MINA predicted poorer OS of the patients who have no lymph node metastasis but better OS of the ones who have signs of lymph node metastasis [24]. The prognostic value of mdig/MINA was also established for the time delay to first progression (FP) in NSCLC patients. High mdig/MINA levels predicted the earlier occurrence of FP of the adenocarcinoma (Ad). However it failed to predict the earlier or later FP of the squamous cell carcinoma. Among those Ad patients, it is interesting to note the opposite predictive power of mdig/MINA for the male and female patients. In male patients, high expression of mdig/MINA is associated with a shortened time to FP, whereas in female patients, high mdig/MINA expression seems to be a favorable factor in prolonging the time to FP [11]. This suggests that mdig/MINA may serve as new marker for predicting disease progression of the lung adenocarcinoma, especially for male patients. Since FP is a life threatening condition, the findings of mdig/MINA's prognostic role on FP may be helpful for clinicians to adopt effective strategies in disease management.

By evaluating mdig/MINA expression through immunohistochemistry of the cancer tissues and the overall survival of 101 lung cancer patients, studies by Komiya et al. [23] revealed that patients who stained negative for mdig/MINA had significantly shorter survival than those who were stained positive for mdig/MINA, especially in stage I or squamous cell carcinoma, indicating that increase of the protein level of mdig/MINA may be associated with a favorable prognosis of lung cancer patients. This appears to be contradictory to what had been found from the analyses of the survival data of 2,437 lung cancer patients as mentioned above. Such a discrepancy can possibly be resulted from the differences in methods to determine the expression of mdig/MINA, the sample size and the ethnic backgrounds of the patient population.

The impact of mdig/MINA on patient survival may be somehow linked to the role of mdig/MINA on cell migration and invasion. In both H226B cells and A549 cells, overexpression of mdig/MINA inhibited cell migration and invasion, whereas silencing mdig/MINA with shRNA enhanced migration and invasion of these cancer cells [13, 23].

#### Neuroblastoma

Mdig/MINA is also one of the prognostic factors in neuroblastoma. A study evaluated two myc gene-regulated proteins, mdig/MINA and Cap 43, in neuroblastoma revealed that mdig/MINA is a clinico-pathological prognostic predicator of neuroblastoma, one of the common pediatric solid cancers. Expression of mdig/MINA was significantly higher in those cases consisting of age more than one year and at an advanced stage. Whereas Cap 43, a metastasis suppressor showed a significant higher expression in those cases consisting of age of less than one year and early stage. There was a positive correlation between Cap 43 and TrkA and a negative correlation between Cap 43 and mdig/MINA, Cap 43 and Ki-67 and mdig/MINA and TrkA, indicating that Cap 43 may be a favorable prognostic factor while mdig/MINA is an adverse prognostic factor in patients suffering from neuroblastoma [31].

#### Colon cancer

High mdig/MINA expression was found in human colon cancer in all the pathological grades and colon tumor cell lines. Mdig/MINA was involved in the proliferation of colon tumor cells and c-myc was the key factor that up-regulate the expression of mdig/MINA in those cells. Suppression of mdig/MINA expression drastically reduced the proliferation of colon tumor cells in vitro [16].

#### Pancreatic cancer

Mdig/MINA is involved in pancreatic ductal carcinoma and there is a relation between the clinico-pathological characteristics of pancreatic cancer and mdig/MINA expression. One study demonstrated that 81 out of 96 (84.4 %) adenocarcinoma specimens showed overexpression of mdig/MINA. A significant association between the histological differentiation, TNM stage and lymph node metastasis with respect to mdig/MINA was found. Silencing of mdig/MINA by siRNA in human pancreatic cell line, PANC-1, induced cell cycle arrest in G2/M phase and apoptosis, resulting in diminished growth of the cells. Thus, mdig/MINA might play an important role in the pancreatic cancer development, as it is overexpressed and is associated with cell proliferation in pancreatic cancer [32].

#### Renal cell carcinoma (RCC)

Mdig/MINA is overexpressed in advanced renal cell carcinoma and its overexpression has an effect on the proliferation of RCC in the late stage and is involved in the dedifferentiation in the advanced stages of RCC. In RCC patients, high mdig/MINA expression correlated with significant shorter survival times. Patients with poor prognostic factors, including stage IV, MVI positivity, sarcomatoid RCC, and high Ki-67 LI, showed high mdig/MINA expression [20].

#### Esophageal squamous cell carcinoma (ESCC)

High mdig/MINA expression level correlates with the poor outcomes of ESCC patients and predicts shorter survival periods in patients suffering from ESCC. RNAi mediated down-regulation of mdig/MINA suppressed cell proliferation of the ESCC cell lines. While mdig/MINA expression was reduced following the suppression of c-myc by c-myc siRNA in cultured ESCC cells, again indicating that mdig/MINA is directly induced by c-myc [17].

#### Lymphoma

Aggressive types of lymphomas frequently display high mdig/MINA expression, such as Burkitt-like lymphoma. MCHL the most aggressive form of Hodgkin's lymphoma, exhibited strongest mdig/MINA expression, followed by an intermediate grade NSHL and with no mdig/MINA expression in indolent LRCHL. Correlation between c-myc and mdig/MINA expression was noted in lymphoma, indicating a possible mutual regulation of these two oncogenic signals in the pathogenesis of lymphomas. Mdig/MINA is highly expressed in Burkitt-like lymphoma, diffuse large B cell lymphoma (DLBCL), and lymphomas with a transition from follicular to DLBCL. In contrast, follicular and T cell lymphomas showed no mdig/MINA expression [18].

#### Gastric carcinoma

Gastric cancers exhibited high expression levels of mdig/MINA and c-myc mRNAs compared to adjacent normal tissues. There was a significant increase in mdig/MINA expression during gastric carcinogenesis that correlated clinico-pathologically with the various factors in intestinal gastric cancer (IGC) and diffuse gastric cancer (DGC) development. Patients with low mdig/MINA has more favorable prognosis compared to those with elevated mdig/MINA expression. Moreover, mdig/MINA expression correlated with that of c-myc expression. There is a strong association between mdig/MINA expression and patient's age, tumor diameter, depth of invasion, distant and lymph node metastasis, and TNM stage in the IGC cases. In DGC category, only lymph node metastasis correlated with high expression of mdig/MINA, suggesting mdig/MINA's participation in lymph node metastasis in DGC. Altogether, high mdig/MINA expression predicted poor prognosis in patients with gastric carcinoma [33].

#### Gingival squamous cell carcinoma (GSCC)

Expression of mdig/MINA and Ki 67 significantly correlated in patients with GSCC. However, non-neoplastic squamous epithelium, dysplasia and GSCC displayed high number of mdig/MINA positive cells than that of Ki 67. Labeling index of mdig/MINA and Ki 67 did not show any significant difference between the well and poorly differentiated GSCC. The predictive value of mdig/MINA in prognosis of GSCC was not observed [34].

#### Cholangiocarcinoma (CCA)

Mdig/MINA is involved in the carcinogenesis of the biliary tract and has clinical significance in CCA. Immunohistochemical analysis showed that 88.4% of the CCA cases exhibited mdig/MINA positivity. Clinico-pathological factors like histological differentiation, TNM stage and lymph node metastasis showed a significant association with the high levels of mdig/MINA expression in CCA, suggesting that mdig/MINA can be used as a marker for CCA. A significant association existed between mdig/MINA and p53 expression as 81.2 % of the CCA samples showed simultaneous upregulation of mdig/MINA and p53. Also increased levels of mdig/MINA were positively associated with Ki-67 levels, hence suggesting that mdig/MINA overexpression is likely to be involved in the proliferation of CCA cells and contribute to CCA development [21].

#### Hepatocellular carcinoma (HCC)

Mdig/MINA may play key roles in the pathogenesis of aggressive hepatocellular carcinoma. Immunohistochemical analysis of mdig/MINA in HCC samples showed diffuse mdig/MINA expression in the nuclei of cancer cells. A strong staining of mdig/MINA was observed at the periphery of the tumor nodules. A significant association exists between mdig/MINA expression and histological grade and poorly differentiated HCC [22].

### Proliferation marker in human cancers: Ki-67 vs mdig/MINA

Ki-67 is a well-used cell proliferation marker and the level of Ki-67 is commonly evaluated in almost all solid tumors as an indicator of the proliferation status among those tumor sub-types. Mdig/MINA is a known oncogene that exerts a proliferative effect in several cancer cell lines and abrogation of mdig/MINA inhibits cell proliferation. In view of this it becomes important and relevant to assess mdig/MINA and Ki-67 status simultaneously in solid tumors. One would expect a similar level of Ki-67 and mdig/MINA expression in the tumor specimens. But this is not always the case. There are several discrepancies in the expression levels of Ki-67 and mdig/MINA in the same sample and tumor subtype.

In lymphomas, mdig/MINA showed a significant correlation with that of Ki-67, suggesting that it is related to cell proliferation in lymphomas. But mdig/MINA is not always expressed in proliferating cells. This is evident by the fact that nearly all the cells in non-neoplastic germinal center exhibit high Ki-67 expression but very faint mdig/MINA in them [18]. Also, in some specimens of esophageal squamous cell carcinoma (ESCC), tumor cells that were strongly stained for mdig/MINA, rarely detected any Ki 67. Though some studies showed mdig/MINA expression was roughly proportional to Ki-67, a considerable number of tissue samples exhibited higher level of mdig/MINA with lower level of Ki-67 [17]. However, the colon and esophageal cancer tissues display close correlation between mdig/MINA and Ki-67 expression, indicating a possible involvement of mdig/MINA in cancer cell proliferation in those tumors. It is not always necessary that the expression of mdig/MINA should coincide with Ki-67 expression and it may not be regarded as carrying out the same function as one contributing to cancer cell proliferation. The evidence supporting this notion is from the fact that very few cells in the basal and parabasal layers of esophageal epithelium displayed Ki-67 expression, whereas many of them exhibit mdig/MINA expression. Moreover, Ki-67 was expressed in the nuclei of mdig/MINA negative colonic cryptic cells [16]. In gingival squamous cell carcinoma (GSCC) that is well differentiated, mdig/MINA expression correlated with that of Ki-67, both being strongly positive in the periphery of the cancer nest. But within the cancer nest, mdig/MINA was expressed in Ki-67 negative cells. In undifferentiated and moderately differentiated GSCC, majority of tumor cells exhibited diffused mdig/MINA expressed throughout while Ki-67 was less densely distributed compared to cells stained positive for mdig/MINA [34].

The possible explanation of the observed discrepancies in mdig/MINA and Ki-67 expression or staining index might owe to the differential expression of mdig/MINA gene and Ki-67 in the different stages of cell cycle. Ki-67 is predominantly expressed in proliferating cells in late G1, S, G2 and M phases but disappear in the cells with a prolonged G1 phase [35-38]. However, mdig/MINA seems to remain expressed even when the cells are under the prolonged G1 phase. Thus, this may facilitate the detection of mdig/MINA in those tumor cells which are in prolonged G1 phase but lack of Ki-67 in the same cell. Moreover, there is a continuous expression of c-myc in the proliferating cells throughout their cell cycle [39, 40] but Ki 67 is selectively expressed in the late G1, S, M and G2 phase of the proliferating cells [35, 36]. Another report suggested that the expression of mdig/MINA is not cell proliferation specific but rather cell type specific. This became evident by the fact that in mouse testis, mdig/MINA was highly expressed in oval shaped cells residing in the periphery of seminiferous epithelium of normal adult testis that is indeed a well proliferating type A spermatogonia. Additionally, highly differentiated cells like sertoli and leydig cells that functions well but are non-dividing, too exhibited mdig/MINA expression [41].

### Environmental insults, mdig/MINA and cancers: an insight

Mdig/MINA is an environmentally induced gene and plays a role in immune system. However, more research is needed to fully elucidate the role of mdig/MINA in mediating immune response. Chronic inflammation has been linked to cancer [42] and is viewed as a critical player in the development of cancers [43]. Since mdig/MINA is a gene that is induced upon exposure to mineral dusts and other environmental factors, the expression of mdig/MINA may be suggestive for different types of cancers associated with environmental and occupational exposure to heavy metals, dusts and smoke. Recent study revealed smoking as a risk factor for breast cancer occurrence [44], and pollutants like diesel exhaust and coal mine dusts predisposes an individual for lung cancer risks in occupational settings [45]. A number of studies suggested the contribution of heavy metal exposure via tobacco smoking, leading to the cancers of head and neck in humans [46]. Individuals working in occupational settings like the one in coal mines and glass industries are constantly exposed to dusts and silica. Inhalation of silica dusts in such settings resulted in the formation of stomach cancers [47, 48], skin cancers [49] bone cancer [50, 51] and esophageal cancer [52, 53]. An extensive body of work exists regarding lung cancer risks with silica exposure [54] and has been reviewed widely [55-57]. Our earlier reports stated that mdig/MINA predicts poorer overall survival in patients with earlier stages of lung cancer [13]. It is interesting to study the underlying reason for this observation. Since mdig/MINA being induced by environmental insults, it becomes obvious to ascertain that mdig/MINA is indeed involved in the development of cancers under occupational settings and exposure to other industrial hazards. Among other environmental contaminants, arsenic is one of the widespread environmental carcinogens found in drinking water, insecticides and herbicides. Over the past several decades, studies on arsenic and human cancer have been well reported [58, 59]. The dose-response relationship between the concentration of arsenic in drinking water and lung cancer incidence is very well documented and a significant correlation occurs between the two [60]. Also, acute exposure to drinking water contaminated with arsenic for the duration of around 5 years resulted in an increased risks for lung cancer [61], whereas significant association exist between people subjected to drinking water with high concentration of arsenic and the increased risks of lung cancers [62, 63]. The evidence that arsenic is highly capable of inducing mdig/MINA in bronchial epithelial cells and lung cancer cells suggests that mdig/MINA is the key mediator for arsenic carcinogenesis [11].

Given the evidence indicating mineral dust exposure and increased risk of cancer development, it is essentially relevant to consider mdig/MINA as an environmental induced gene that contributes to development of lung cancer, breast cancer, oral cancer, and cancers of the gastrointestinal tract. Animal models of human cancers lacking mdig/MINA gene or overexpressing it, should be generated to better understand and decode the function of mdig/MINA in orchestrating the early events of neoplasia, cancer progression and metastasis. This will enhance our existing knowledge on mdig/MINA in human cancers and would lead to the discovery of more prominent novel bio-molecules of diagnostic and therapeutic importance in human malignancies.

### Mdig/MINA, arsenic and silica exposure: mechanistic insight

We have already shown that hazardous environmental agents like arsenic and silica are able to induce the expression of mdig/MINA in lung epithelial cells as well as lung cancer cells [2, 11]. Most recently we evaluated particulate matter PM 2.5 for its ability to induce mdig/MINA in human bronchial epithelial cell line BEAS-2B (unpublished results). At present, it remains to be fully elucidated that how these environmental factors induce mdig/MINA. Since the first discovery of mdig/MINA gene, we have been dissecting the role of mdig/MINA in cell proliferation, epigenetics, immune regulation, and malignancy using normal lung cells, lung cancer cell lines and mouse models. Towards this direction, we have found that arsenic induces the expression of mdig/MINA through JNK and STAT3 signaling pathways, with microRNA-21 (miR-21) and AKT acting as the downstream effectors [11]. In other studies, we had explored the possible molecular mechanisms of arsenic-induced carcinogenesis using normal bronchial epithelial cells BEAS-2B and found that arsenic induces the phosphorylation of EZH2 at serine 21 and this phosphorylation of EZH2 requires arsenic-activated signaling cascade from JNK and STAT3 to AKT [25]. EZH2 is the enzymatic subunit of polycomb repressive complex 2 (PRC2) that is responsible for promoting the tri-methylation of H3K27me3, leading to altered expression of tumor suppressors or oncogenes. We had also reported the involvement of reactive oxygen species (ROS) in arsenic induced activation of JNK-STAT3-AKT signaling axis that is linked to EZH2 phosphorylation [64]. Furthermore, we had shown that in response to arsenic, AKT dependent filamin A phosphorylation promotes cell migration. Filamin A, a cytoskeleton remodeling protein, may be involvement in arsenic-induced carcinogenesis. This notion is supported by the fact that in lung cancer patients with adenocarcinoma, increased expression of filamin A predicted poorer overall survival [65]. Thus, it is very likely that protein kinases, EZH2 and filamin A may converge together for the expression of mdig/MINA in response to arsenic as well as other environmental hazards. To further explore how environmental factors induce mdig/MINA and how mdig/MINA executes its effect on environmental factor-mediated carcinogenesis, we generated mdig/MINA gene knockout mice. We found that mdig/MINA is essential for silica-induced lung fibrosis and its deficiency ameliorates the fibrotic process by altering the balance between Th17 and Treg cells in the lung [30]. We anticipate that our research on mdig/MINA gene and its role in carcinogenesis will provide significant information on how mdig/MINA is induced by environmental factors and whether mdig/MINA can serve as a key player in environmental factor-induced diseases and cancers.

### Perspectives: targeting mdig/MINA for therapeutics

**Figure 4:**
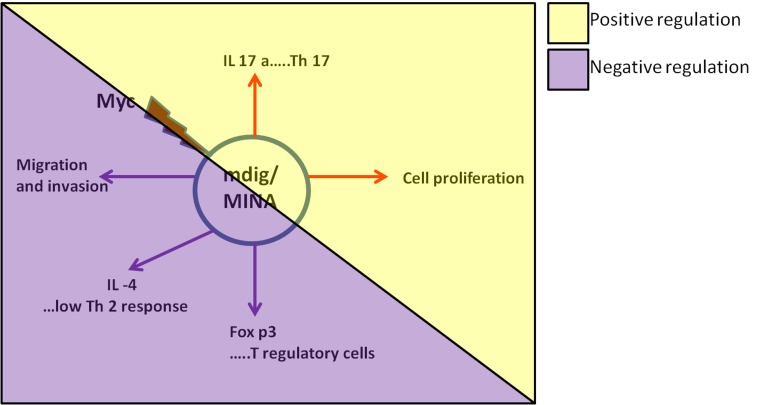
Role of c-myc-targeting gene mdig/MINA in cell growth and immune system Paradoxical role of mdig/MINA in cell proliferation, motility and invasion. It promotes cell proliferation whereas exerts inhibitory effects on migration and invasion. In the immune system, mdig/MINA positively regulates Th17 differentiation while inhibits Foxp3 programs shutting the production of Treg cells. It negatively regulates the IL-4 production thereby reducing the Th2 cell-mediated immune response.

Oncogene c-myc is a widely studied proto-oncogene whose overexpression has been associated with a variety of human cancers. There is evidence suggesting that c-myc is the key transcription factor for the expression of mdig/MINA that is found to be elevated in human cancers with poor prognosis. It becomes important to dissect how c-myc modulates mdig/MINA or how mdig/MINA regulates other major known or unknown genes/players in the neoplastic transformation. Mdig/MINA's involvement in cell proliferation suggests its contribution to the initial process of carcinogenesis, which may render it as an important factor in regulating the early events of tumor formation. Interestingly, mdig/MINA's inhibitory effect on cell motility and invasion makes it a critical player in orchestrating the metastasis of the tumor cells (Figure 4). Recently, studies performed in heterozygous myc knockout mice (myc+/−) revealed that decreased myc expression promoted longevity and benefited multiple organs and physiological process thereby enhancing the health span of the animals. Age-related pathologies, notably cardiac fibrosis and immunosenescence were found to be attenuated in the myc+/− mice [66]. This is an important finding that further strengthens our mdig/MINA knockout studies reporting that mdig/MINA deficiency ameliorated silica-induced pulmonary fibrosis in mice by altering the balance between Th17 and Treg cells. Similar to the myc+/− mice, we observed that the mdig/MINA knockout mice were perhaps healthier and lived a bit longer compared to their wild type counterparts [30]. Since mdig/MINA promotes Th17 response and inhibits Treg cells (Foxp3 positive) [27], disrupting the mdig/MINA's activity/levels is likely to affect the Th17 response. It would be interesting to explore the possibility that Th17 cells may serve as a down-stream effector of mdig/MINA in carcinogenesis or tumorigenesis induced by environmental risk factors.

**Figure 5:**
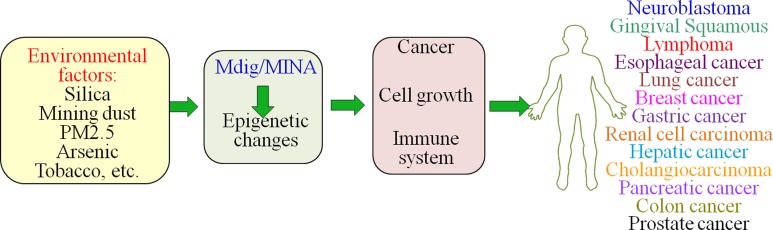
Multifaceted roles of mdig/MINA as summarized by the cell culture studies, murine experiments and data from human cancers Exposure to environmental and occupational hazards constantly puts risks of cancer development in individuals. Mdig/MINA is an environmentally-induced gene that plays an important role in cell proliferation, migration/invasion and immune function, possibly through epigenetic regulation on the genome.

Currently we are trying to understand the interaction of mdig/MINA with various moieties in context to cell transformation. In our most recent proteomic analyses, we found that mdig/MINA is able to interact with several chromatin binding proteins, such as XRCC5, RBBP4/7, CBX8, TDRD, etc. [67]. Accordingly, we believe that mdig/MINA may accomplish its function on epigenetic regulation, DNA replication, DNA repair, and cell growth through interacting with other proteins. In human multiple myeloma cells, we also found overexpression of mdig/MINA is associated with disease onset, progression and relapse. High level of mdig/MINA is an indicator for poor diagnosis of multiple myeloma, possibly due to the enhanced Jak-STAT signaling by mdig/MINA (Wu et al. in preparation). Considering the fact that mdig/MINA is an environmentally induced gene that orchestrates several steps leading to human cancers (Figure 5), developing therapeutic chemicals or reagents that target mdig/MINA will be beneficial for cancer treatment in clinics.
